# Supplementary material for: First Dye-Decolorizing Peroxidase from an Ascomycetous Fungus Secreted by Xylaria grammica
Source: Biomolecules. 2021 Sep 21;11(9):1391. doi: 10.3390/biom11091391 (PMC8469222; doi:10.3390/biom11091391)

Supplementary Material

S1 Proteomics (Peptide mapping)

Each sample was prepared for proteolytic cleavage before mass spectrometric analysis. Protein lysate was reduced (2.5 mM DTT for 1 h at 60 °C) and alkylated (10 mM iodoacetamide for 30 min at 37 °C). Proteolysis was performed overnight using trypsin (Promega, Madison, WI, USA) with an enzyme/substrate ratio of 1:25 overnight at 37 °C by trypsin (Promega). Extracted peptide lysate was desalted using a C_{18} ZipTip column (Merck Millipore).

Peptide lysates were dissolved in 0.1 % formic acid and injected into liquid chromatography mass spectrometry (LC-MS/MS) apparatus. Mass spectrometry was performed on a Q Exactive HF MS (Thermo Fisher Scientific, Waltham, MA, USA) with a TriVersa NanoMate (Advion, Ltd., Harlow, UK) source in LC chip coupling mode. First, the peptide lysates were separated on a UHPLC system (Ultimate 3000, Dionex/Thermo Fisher Scientific, Idstein, Germany). In total, 5 µL samples were first loaded for 5 min on the precolumn (µ-precolumn, Acclaim PepMap, 75 µm inner diameter, 2 cm, C18, Thermo Scientific) at 4 % mobile phase B (80 % acetonitrile in nanopure water with 0.08 % formic acid), 96 % mobile phase A (nanopure water with 0.1 % formic acid), then eluted from the analytical column (PepMap Acclaim C18 LC Column, 25 cm, 3 µm particle size, Thermo Scientific) over a 90 min non-linear gradient of mobile phase B (4-55 % B).

The mass spectrometer was set on loop count of 20 used for MS/MS scans with higher energy collision dissociation (HCD) at normalized collision energy of 30 %. MS scans were measured at a resolution of 120,000 in the scan range of 350-1,550 *m/z*. MS ion count target was set to 3×10^6 at an injection time of 80 ms. Ions for MS/MS scans were isolated in the quadrupole with an isolation window of 1.6 Da and were measured with a resolution of 15,000 in the scan range of 200-2,000 *m/z*. The dynamic exclusion duration was set to 30 s with a 10 ppm tolerance. Automatic gain control target was set to 2×10^5 with an injection time of 120 ms using the underfill ratio of 1 %.

The software Proteome Discoverer (v1.4, Thermo Scientific) was used for protein identification. Therefore, the measured MS/MS spectra (*.raw files) were searched with the Sequest HT algorithm first against the Uniprot (bacteria and archaea database). The following settings were selected for both searches: enzyme specificity was trypsin with up to two missed cleavages allowed using 10 ppm peptide ion tolerance and 0.02 Da MS/MS tolerances. Oxidation (methionine) and acetylation (any N-terminus) were selected as a dynamic modification and carbamidomethylation (cysteine) as a static modification. Only peptides with a false discovery rate (FDR) <0.01 calculated by Percolator, peptide rank =1 and XCorr >2 were considered as identified.

Table S1. Summary of the properties of the three genes encoding DyPs, which were identified in the *X. grammica* genome (bold letters indicate the gene belonging to the purified *Xgr*DyP).

Gene	ORF	Introns	Protein	Mw (kDa)	pI	Signal peptide	Glycosylation sites
**488**	**1,888**	**6**	**493**	**53.9**	**6.45**	**-**	**2**
1956	3,642	1	1,196	133.0	5.16	-	2
9177	1,095	0	364	40.4	6.73	-	3

Table S2. Kinetic cobnstants (K_M (µM), k_{cat} (s^{-1}) and k_{cat}/K_M (s^{-1} mM^{-1}) for the oxidation of ABTS, 2,6-DMP, RBlue 5 and Mn^{2+} by basidiomyceteous wild-type and recombinant DyPs of *A. auricula judae* (*Aau*DyP1 & 2), *Pleurotus sapidus* (r*Psa*DyP) and *P. ostreatus* (r*Pos*DyP1 & 4) in comparison to recombinant bacterial DyPs from *P. fluorescens* (r*Pfl*DyP1B & r*Pfl*DyP2B) and *R. jostii* (r*Rjo*DyPA) as well as to wild-type ligninolytic MnPs (*Pos*MnP3 & 6) & VPs (*Pos*VP1 & 2) of *P. ostreatus*; references are given in parentheses.

Substrate		*Aau*DyP1 [1]	*Aau*DyP2 [1]	r*Psa*DyP [2]	r*Pos*DyP1 [3]	r*Pos*DyP4 [3]	r*Pfl*DyP1B [4]	r*Pfl*DyP2B [4]	r*Rjo*DyPA [5]	*Pos*MnP3 [3]	*Pos*MnP6 [3]	*Pos*VP1 [3]	*Pos*VP2 [3]
ABTS	K_M	18	20	99	779	787	1,130	1,700	8,200	778	1,020	4	495
	k_{cat}	292	509	375	208	277	13.5	10.2	17	222	115	14	12
	k_{cat}/K_M	16,222	25,450	3,788	267	352	11.9	6	2	285	112	3,600	9
2,6-DMP	K_M	27	23	1,227	31,100	126				59,100	117,000	54	607
	k_{cat}	90	100	60	64	268				101	56	7	17
	k_{cat}/K_M	3,333	4,348	50	2.1	2,120				1.7	0.5	122	28
RBlue5	K_M	-	-	24	-	5.7				-	-	5.4	9.6
	k_{cat}	-	-	18	0	5.3				0	0	12.9	20.3
	k_{cat}/K_M	-	-	750	0	1,080				0	0	2,380	2,120
Mn^{2+}	K_M	-	-		2,780	286	7,300	1,700	24,000	101	73	98	18
	k_{cat}	-	-		10	56	2.4	3.6	0.6	163	109	185	79
	k_{cat}/K_M	-	-		4	196	0.33	2.1	0.02	1,010	1,500	1,900	4,510

1. Bobeth, C.: Produktion, Reinigung und Charakterisierung einer extrazellulären Peroxidase des Weissfäulepilzes *Auricularia auricula-judae* (Judasohr). Bachelor Thesis, International Institute Zittau, Germany, 13.10.2008
2. Fernández-Fueyo, E.; Linde, D.; Almendral, D.; López-Lucendo, M.F.; Ruiz-Dueñas, F.J.; Martínez, A.T. Description of the first fungal dye-decolorizing peroxidase oxidizing manganese(II). *Appl Microbiol Biotechnol* **2015**, *99*, 8927-8942.

3. Lauber, C., Schwarz, T., Nguyen, Q.K., Lorenz, P., Lochnit, G., Zorn, H. Identification, heterologous expression and characterization of a dye-decolorizing peroxidase of *Pleurotus sapidus*. *AMB Expr* **7,** 164, https://doi.org/10.1186/s13568-017-0463-5
4. Rahmanpour, R.; Bugg, T.D.H. Characterisation of DyP-type peroxidases from *Pseudomonas fluorescens* Pf-5: Oxidation of Mn(II) and polymeric lignin by Dyp1B. *Arch Biochem Biophys* **2015**, *574*, 93-98.
5. Roberts, J.N.; Singh, R.; Grigg, J.C.; Murphy, M.E.; Bugg, T.D.; Eltis, L.D. Characterization of dye-decolorizing peroxidases from *Rhodococcus jostii* RHA1. *Biochemistry* **2011**, *50*, 5108-5119.

Figure S1. Protein sequence of the *X. grammica* DyP encoded by the gene *g488*; grey highlighted parts are internal *de-novo* peptide sequences of the purified protein identified by mass spectrometry.

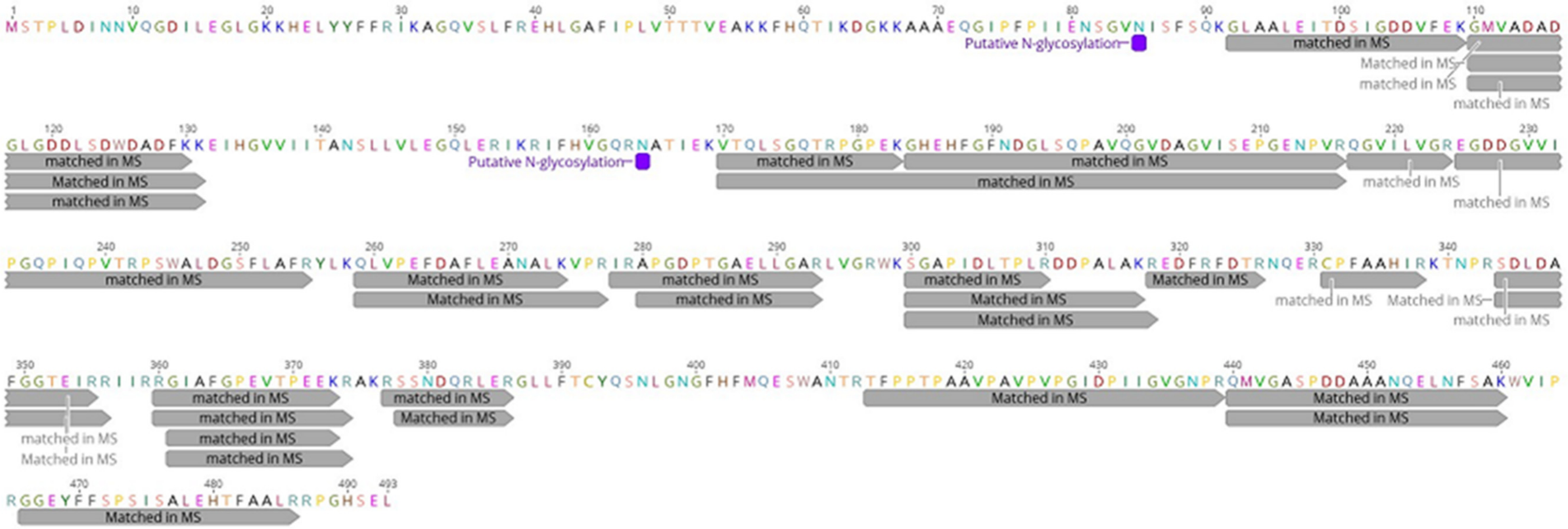

Figure S2. Complete alignment of characterized fungal DyPs with sequences obtained from the genome of *X. grammica* (*g9177*, *g1956* and *g488*); conserved proximal and distal residues (red) responsible for Fe-binding (blue) as well as H_2O_2-binding (F & L) or acting as acid base catalysts (D & R).

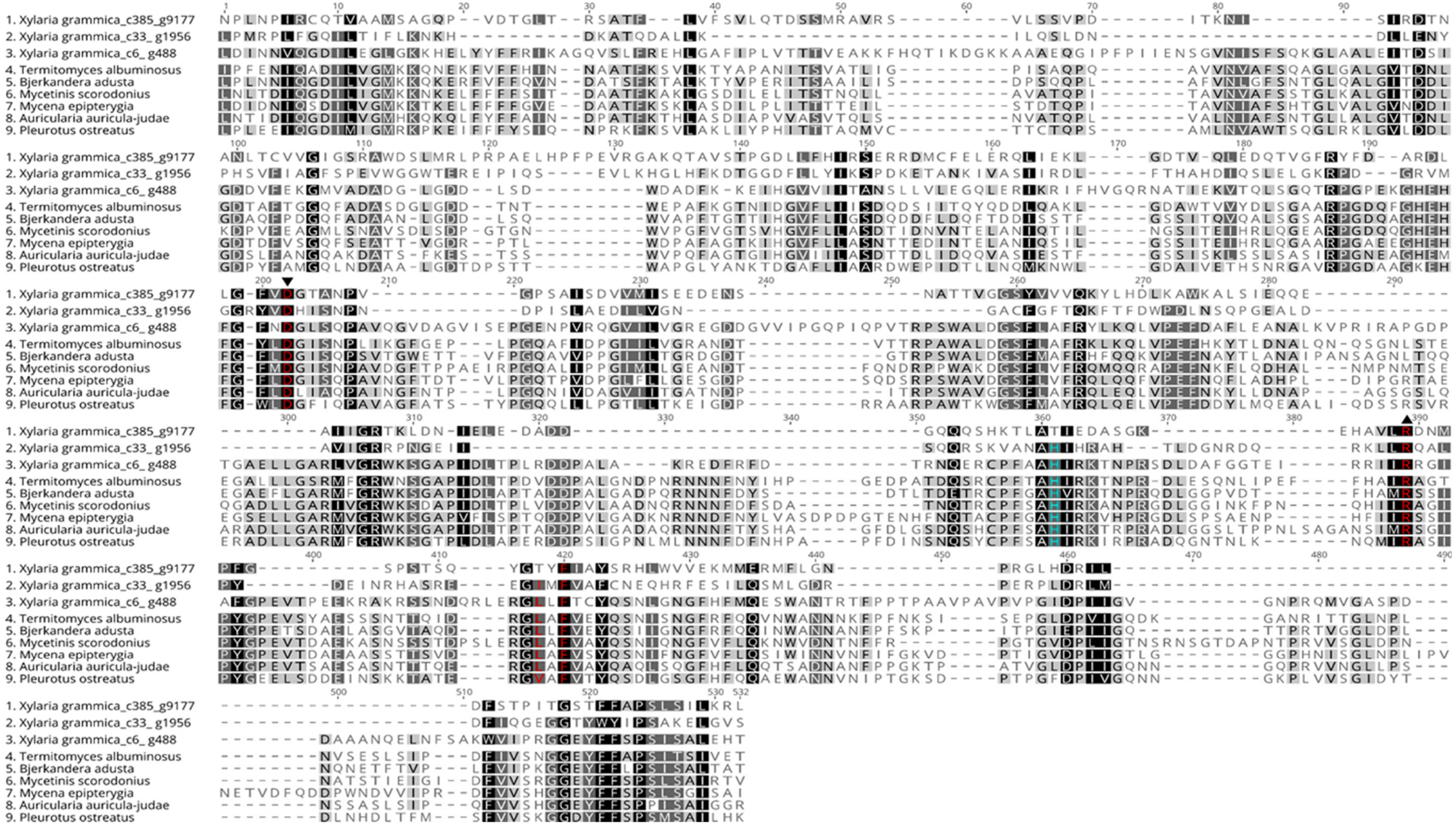

Figure S3. Conserved residues of the heme binding site of three *X. grammica* DyPs in comparison to other bacterial and fungal DyP-type peroxidases.

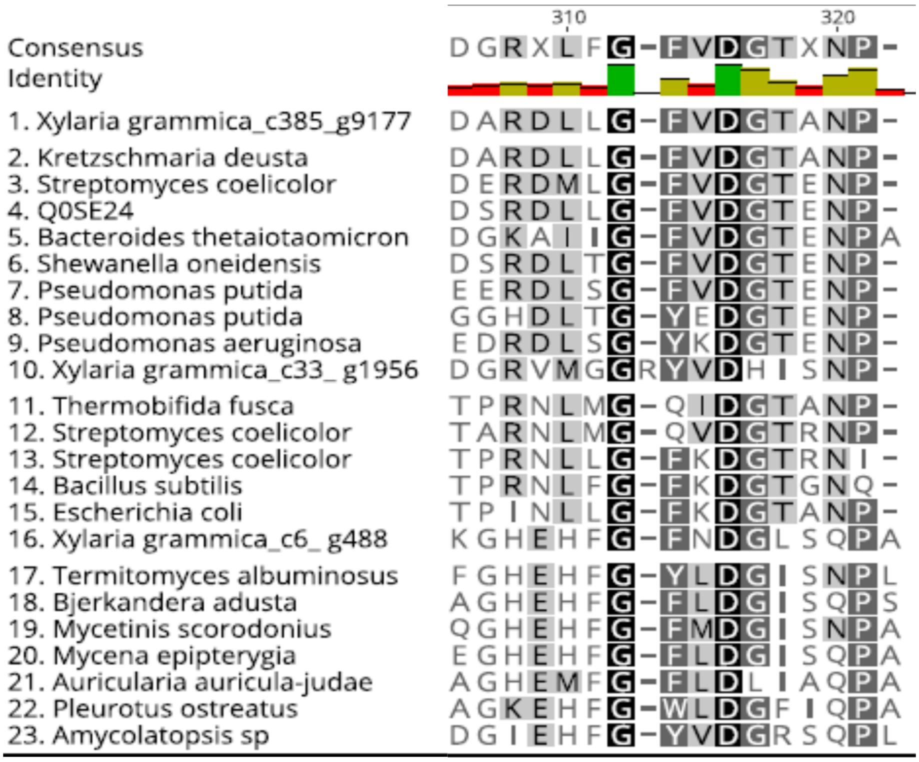

Supplement: Supplementary file 1 [file biomolecules-11-01391-s001.zip › biomolecules-1352879-SI.pdf]
